# An Overview of Linear Dielectric Polymers and Their Nanocomposites for Energy Storage

**DOI:** 10.3390/molecules26206148

**Published:** 2021-10-12

**Authors:** Lvye Dou, Yuan-Hua Lin, Ce-Wen Nan

**Affiliations:** 1 State Key Laboratory of New Ceramics and Fine Processing, School of Materials Science and Engineering, Tsinghua University, Beijing 100084, China; lvyedu1102@tsinghua.edu.cn (L.D.); cwnan@tsinghua.edu.cn (C.-W.N.); 2 Foshan (Southern China) Institute for New Materials, Foshan 528000, China

**Keywords:** linear dielectric polymers, nanocomposites, energy storage capacitor, discharge density, efficiency

## Abstract

As one of the most important energy storage devices, dielectric capacitors have attracted increasing attention because of their ultrahigh power density, which allows them to play a critical role in many high-power electrical systems. To date, four typical dielectric materials have been widely studied, including ferroelectrics, relaxor ferroelectrics, anti-ferroelectrics, and linear dielectrics. Among these materials, linear dielectric polymers are attractive due to their significant advantages in breakdown strength and efficiency. However, the practical application of linear dielectrics is usually severely hindered by their low energy density, which is caused by their relatively low dielectric constant. This review summarizes some typical studies on linear dielectric polymers and their nanocomposites, including linear dielectric polymer blends, ferroelectric/linear dielectric polymer blends, and linear polymer nanocomposites with various nanofillers. Moreover, through a detailed analysis of this research, we summarize several existing challenges and future perspectives in the research area of linear dielectric polymers, which may propel the development of linear dielectric polymers and realize their practical application.

## 1. Introduction: Basic Knowledge of Dielectric Capacitors

**Figure 1:**
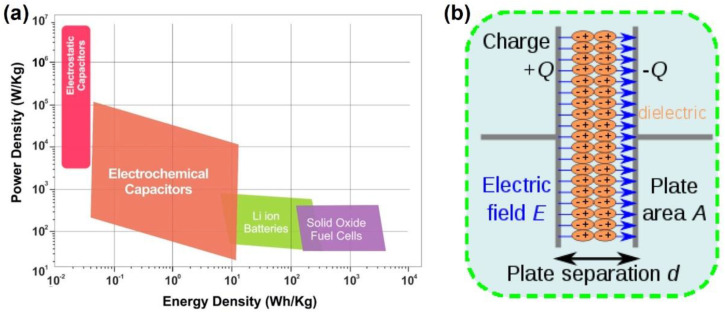
(**a**) Sketch of a Ragone plot for various energy storage device systems. Reproduced with permission from Ref. [11]. Copyright 2017, American Institute of Physics. (**b**) The diagram of charge separation in a parallel-plate capacitor under the function of an electric field. Reproduced with permission from Ref. [1]. Copyright 2013, World Scientific.

The ever-increasing development of new energy generation technologies has led to higher requirements for the development and performance improvement of energy storage devices [1]. To date, the most commonly used energy storage devices mainly include dielectric capacitors [2,3], electrochemical capacitors [4,5], batteries [6,7], and fuel cells [8,9]. Among them, dielectric capacitors are competitive due to their ultrahigh power density [10]. However, compared with electrochemical capacitors and batteries, dielectric capacitors usually exhibit an ultralow energy density, as shown by the Ragone plot presented in Figure 1a [11]. As a result, dielectric capacitors are used for critical applications in various electrical systems but their low energy density significantly limits the miniaturization of devices [12,13]. Therefore, the energy storage performance of dielectrics must be significantly improved to enable their extensive practical application [14,15].

Figure 1b shows the basic structure of a dielectric capacitor: a middle layer containing a dielectric material, with a conductive plate (electrode) on each side [1,16]. Once an electric field, E, is applied to the capacitor, the dielectric material between the electrodes is quickly polarized. Therefore, positive and negative charges of the same amount are separately accumulated on the two electrodes. The energy storage ability of a dielectric capacitor is defined as capacitance (*C*), which is usually expressed as shown in Equation (1).

(1) $$C=\varepsilon_{r}\varepsilon_{0}\frac{A}{d}$$

In Equation (1), *ε*_r_ represents the permittivity of a dielectric material, and the value of *ε*_r_ is determined by its intrinsic properties. Thus, *ε*_0_ is the vacuum permittivity, which is a constant value of ~8.85 × 10^−12^ F/m. *A* and *d* are the area of the electrode and the distance between the two electrodes (the thickness of the dielectric material), respectively. *C* is directly proportional to *ε*_r_ and *A*, and it is inversely proportional to *d* [1,2].

The capacitance *C* can also be defined according to the incremental change of the charge (*Q*) with respect to voltage (*V*) and defined geometrically using the following equation:

(2) $$C=\frac{d_{Q}}{d_{V}}$$

In this equation, *V* is the external voltage applied to the two conductive plates and *Q* represents the equal positive and negative charges accumulated on both conductive plate surfaces. Therefore, the energy stored in a dielectric material (*W*) can be expressed as:

(3) $$W=\int_{0}^{Q_{\max}}Vd_{Q}$$

The charge density (*Q*/*A*) on the plate surfaces equals the electric displacement *D* (where *D* = *ε*_0_ *ε*_r_ *E*) in the dielectric material. Therefore, according to Equation (3), the energy density *J* can be expressed by the following equation [17]:

(4) $$J=\frac{W}{Ad}=\frac{\int_{0}^{Q}Vd_{Q}}{Ad}=\int_{0}^{D_{\max}}EdD$$

where *E* is the external applied electric field (equal to *V*/*d*) and *D*_max_ is the electric displacement under the field *E*_max_. For dielectrics with high permittivity, the electric displacement *D* is very close to the electric polarization *P*. Thus, Equation (4) can be rewritten as:

(5) $$J=\int_{0}^{P_{\max}}EdP=\int_{0}^{E_{\max}}\varepsilon_{0}\varepsilon_{r}EdE$$

**Figure 2:**
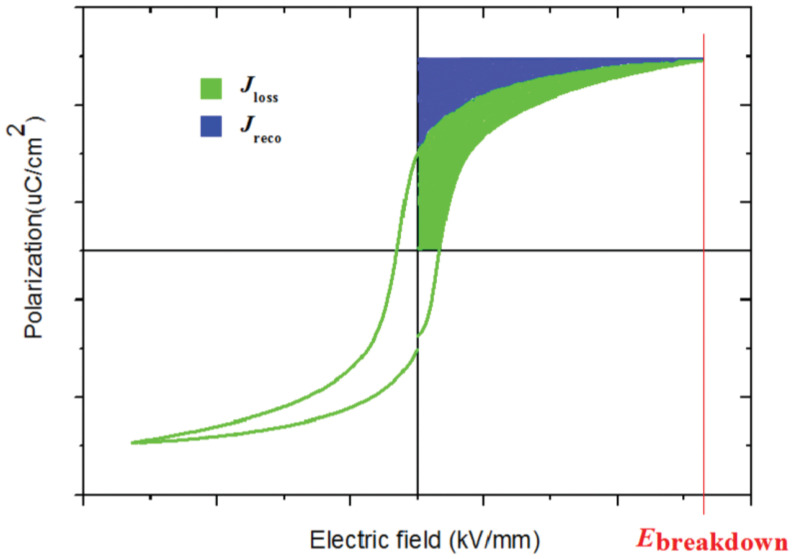
Graphical representation of a *P*–*E* loop used for energy-storage calculations. Reproduced with permission from Ref. [18]. Copyright 2017, Wiley–VCH.

By combining Equation (5) with the *P*–*E* curve shown in Figure 2, the value of *J* can be obtained by integrating the curves of the *P*–*E* loop [18,19]. Thus, the blue-colored region in Figure 2 represents the recoverable energy density (*J*_reco_), while the green shaded region represents the energy density dissipated during the discharge process (*J*_loss_) [18]. The *J*_reco_ can be improved in two ways: by enhancing the breakdown strength and increasing the polarization. 

Generally, *J*_loss_ is caused by leakage and polarization hysteresis. On this basis, the charge-discharge efficiency *η* is defined as:

(6) $$\eta =\frac{J_{\mathrm{reco}}}{J_{\mathrm{reco}}+J_{\mathrm{loss}}}\times 100\%$$

For linear dielectrics, the dielectric constant is independent of the electric field. Thus, the energy density of linear dielectrics is expressed by Equation (7):

(7) $$J=\frac{1}{2}\varepsilon_{0}\varepsilon_{r}E^{2}$$

where the value of *J* is proportional to the value of *ε*_r_ and the square of *E* [11,18].

## 2. Classification of Dielectric Materials

### 2.1. Four Typical Dielectric Materials

**Figure 3:**
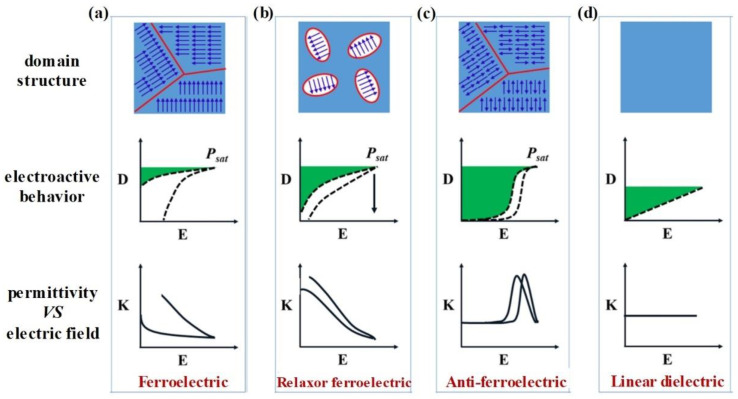
Schematic showing the typical classification of dielectric materials (**a**) Ferroelectric, (**b**) Relaxor ferroelectric, (**c**) Anti-ferroelectric, (**d**) Linear dielectric. Reproduced with permission from Ref. [21]. Copyright 2018, Elsevier.

As the core component of dielectric capacitors, the intrinsic characteristics of dielectrics have a great impact on the energy storage performance of the capacitor. Dielectric capacitors will only be widely used in practical applications if they can exhibit high recoverable energy storage density and efficiency. To achieve this goal, three basic requirements need to be satisfied: high breakdown strength, high saturation polarization, and low remnant polarization [20]. To date, four typical dielectrics (ferroelectric, relaxor ferroelectric, anti-ferroelectric, and linear dielectrics) have been widely studied. The domain structure, electroactive behavior, and permittivity vs. the electric field of these dielectric materials are presented in Figure 3 [21]. 

As Figure 3a demonstrates, the D–E curves of ferroelectrics during the charging–discharging process exhibit remarkably nonlinear characteristics, and the permittivity shows a decreasing tendency with the increasing electric field [21,22]. In addition, ferroelectrics often exhibit high remnant polarization (*P_rem_*), leading to a typical rectangular-shaped hysteresis loop, as can be seen from the electroactive behavior of ferroelectrics, shown in Figure 3a. The apparent hysteresis loops in ferroelectrics are mainly caused by the switching of large ferroelectric (FE) domains [23,24]. Overall, ferroelectrics usually exhibit relatively high energy densities because of the relatively high saturation polarization (*P_sat_*) and moderate breakdown strength. However, the high *P_rem_* values result in low efficiency. 

Compared to ordinary ferroelectrics, the nanodomain structure of relaxor ferroelectrics is more disordered. The dispersion distribution of their structure and polarization leads to a dispersion distribution of macroscopic dielectric properties. Due to their small domain size and low energy barriers, these nanodomain structures are easily disturbed by thermal excitation or by flipping the electric field [25]. In addition, coupling between the domains is weak and the energy consumption of domain inversion is reduced, leading to a relatively low remnant polarization. As a result, low loss (and thus high efficiency) is achieved while maintaining high polarization, leading to a narrow hysteresis loop (Figure 3b).

As shown by Figure 3c, the electric behavior of antiferroelectrics is quite different from that of ferroelectrics and relaxor ferroelectrics. Specifically, the *P_rem_* of antiferroelectrics is zero, and the D–E curves of antiferroelectrics during the charging and discharging process display a double hysteresis loop [1]. Accordingly, the permittivity of antiferroelectrics remains constant at a low electric field but if the electric field increases, the permittivity first sharply increases and then rapidly decreases. The distinctive energy storage behavior of antiferroelectrics is mainly due to their unique domain structure. As presented in Figure 3c, the spontaneous polarization directions of adjacent dipoles in antiferroelectrics alternate in opposite directions, although polarization direction can be induced to have the same orientation under the function of an electric field [26,27].

Unlike the other three typical dielectric materials, linear dielectrics exhibit a linear D−E loop, and their permittivity remains constant under an increasing electric field (Figure 3d), which is mainly due to the lack of an FE domain [21,23]. Therefore, linear dielectrics generally possess a high electric breakdown strength with almost no energy loss, making them suitable for use in high-efficiency capacitors. However, linear dielectrics usually exhibit a relatively low permittivity. For example, the permittivities of commercial biaxially oriented polypropylene (BOPP) [28], polyetherimide (PEI) [29], and polyethylene terephthalate (PET) [30] are 2.2, 3.2, and 3.3, respectively. Therefore, the energy density of these materials will be significantly improved with the increase of the dielectric constant. Based on this analysis, a significant amount of research has been carried out, and select detailed methods will be discussed in Section 3 of this review.

### 2.2. Linear Dielectric Polymers

In the early development of dielectric capacitors, piezoelectric ceramics were regarded as the most promising dielectric materials due to their high dielectric constants [31]. However, these ceramics usually exhibit low breakdown strength, making them unfavorable for improving capacitor energy density. In addition, the practical application of ceramics is severely limited by their poor flexibility and high density [32]. In contrast to ceramic dielectrics, polymer dielectrics usually exhibit higher breakdown strength, better flexibility, and improved ease of processing [33]. Therefore, polymer dielectric materials are more suitable for the fabrication of dielectric capacitors with improved energy storage performance [24,34,35].

As mentioned in Section 2.1, linear dielectrics exhibit nearly linear D–E loops, due to their field-independent dielectric constant. Because of this, linear dielectrics usually exhibit higher efficiency than ferroelectrics. Therefore, in the past decades, researchers have carried out a significant amount of research on linear polymer dielectrics. In this section, several typical linear dielectric polymers will be briefly introduced. 

#### 2.2.1. BOPP

Currently, BOPP is one of the most commonly used commercial dielectrics, due to its excellent breakdown strength and the low dielectric loss of polypropylene (PP) [36]. PP is a typical linear dielectric polymer that exhibits nearly frequency-independent and temperature-independent permittivity. Therefore, BOPP films prepared by melt extrusion and the biaxial stretching of PP show high efficiency. However, due to the intrinsic low permittivity of PP, the BOPP usually exhibits a low energy density (<2 J/cm^3^) [28]. In addition, the working temperature of BOPP is usually below 105 °C, which significantly limits its application in high-temperature environments [37,38]. 

#### 2.2.2. Polyimide (PI) 

In addition to BOPP, PI is another important linear dielectric polymer. The thermal stability of PI (>500 °C) is much higher than that of BOPP (<105 °C), which is mainly due to the imide structure in its main chain. In addition, PI also exhibits good mechanical and chemical resistance, as well as excellent dielectric properties, as summarized in Table 1 [38,39]. Therefore, several kinds of commercial PIs, such as Kapton (developed by DuPont) and UPILEX (developed by UBE), have been developed for high-performance dielectric capacitors. However, the conduction loss of PI rapidly increases with increasing temperature. Specifically, at 150 °C, the conduction loss of Kapton under 200 MV/m is as high as 24% (i.e., the efficiency is only 76%), and the conduction loss further increases to almost 100% at 250 °C [40]. 

#### 2.2.3. PEI

PEI is an amorphous engineering thermoplastic dielectric polymer, with similar dielectric properties to those of PI. Compared with PI, the molecular chain of PEI has flexible ether linkages, improving the processability of PEI. However, PEI exhibits poor thermal stability compared to PI [38]. For example, ULTEM (developed by SABIC) is one of the most important commercial PEI products but its glass transition temperature (*T*_g_) ranges from 217 °C to 247 °C, much lower than that of Kapton or UPILEX. However, compared with PI, PEI exhibits better energy storage performance under high temperatures (up to 200 °C). For instance, at 150 °C, PEI has an efficiency of up to 90% under 200 MV/m. The efficiency of PEI still approaches 80% at 200 °C, exhibiting much better high-temperature energy storage performance than PI [41].

#### 2.2.4. Other Linear Dielectric Polymers

In addition to BOPP, PI, and PEI, many other polymers, such as low-density polyethylene (LDPE) [42], poly (arylene ether urea) (PEEU) [43], polymethyl methacrylate (PMMA) [44], polyethylene terephthalate (PET) [37], and polycarbonate (PC) are also linear dielectric polymers [45]. Table 1 summarizes some of the basic properties of these linear dielectric polymers. 

### 2.3. Linear Dielectric Polymer Blends

Although the low permittivity of linear dielectrics usually limits an increase in their energy density, their clear superiority in charge–discharge efficiency compared to other dielectric materials has attracted an increasing amount of attention. To date, several reports dedicated to increasing the energy density of linear dielectrics have been published [46,47,48,49]. Most of these reports mainly focus on improving the permittivity or breakdown strength of dielectrics by molecular structure design or component regulation.

As is well known, the dielectric breakdown strength of polymers is closely related to the weak points in dielectric materials. Generally, the free volume between molecular chains, physical voids, and disordered structures in materials are all dielectric defects [50,51,52]. Therefore, it is necessary to minimize these micro- and macrostructural defects in dielectric materials. Based on this analysis, researchers have effectively reduced the microstructural defects in polymers by reducing the free volume between molecular chains [53]. In brief, PI/PEI composite films with reduced weak points were obtained by a feasible blending strategy. 

**Figure 4:**
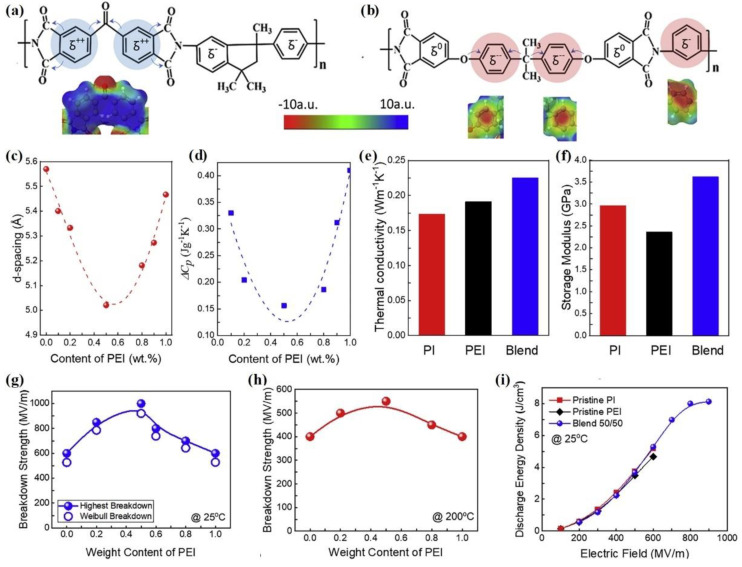
Schematic chemical structures of PI (**a**) and PEI (**b**). (**c**,**d**) Average interchain spacing (**c**) and the change in specific heat capacity during glass transition (*ΔC_p_*) (**d**) for PI/PEI blends with different ratios. (**e**,**f**) Thermal conductivity (**e**) and storage modulus (**f**) of pristine PI, PEI, and PI/PEI blend with a 50/50 ratio. (**g**,**h**) Dielectric breakdown strength as a function of the PI/PEI blend ratio at room temperature (**g**) and at 200 °C (**h**). (**i**) Discharge energy density of PI/PEI blend films under various electric fields at room temperature. Reproduced with permission from Ref. [53]. Copyright 2021, Elsevier.

Figure 4a presents the schematic chemical structure of PI, which shows two positively charged phenyls. In contrast, the molecular chain of PEI exhibits significant electronegativity, as shown in Figure 4b. Therefore, the packing density and defects of molecular chains can be controlled by adjusting the ratio of PI/PEI (wt%/wt%). Figure 4c presents the average interchain spacing of PI/PEI dielectric films as a function of PEI content [53]. The results showed that PI/PEI blends, with a ratio of 50/50, exhibited the smallest interchain spacing, 10% lower than that of unadulterated PI or PEI. Similarly, Zhang et al. calculated the change in the specific heat capacity of blended films with varying PEI content during glass transition (Figure 4d), and their results were consistent with those of Figure 4a. Thus, PI/PEI blended films with 50% PEI content exhibited the most extended polymer chains among all the reported samples [54,55,56,57]. Furthermore, PI/PEI blend films with 50% PEI content also exhibited a higher density (1.35 g/cm^3^) compared to both pure PI film (1.3 g/cm^3^) and pure PEI film (1.27 g/cm^3^). Meanwhile, the thermal conductivity and storage modulus values of PI/PEI blended films with 50% PEI content were also higher than the values of PI and PEI (Figure 4e,f). In conclusion, this article presented a general strategy to reduce the weak points, such as voids and free volume in polymers by exploiting interchain electrostatic forces in polymer blends. The reduced weak points could be attributed to the dense chain packing in the blend polymers. Therefore, modifying the polymer molecular chain to reduce structural defects is an important method to improve the breakdown strength of linear polymers.

The micro-structured weak points in PI/PEI blended films decrease in number due to the reduced interchain spacing, significantly improving their breakdown strength. Figure 4g presents the breakdown strength of various PI/PEI blended films at room temperature. As can be seen, PI/PEI films with 50% PEI content exhibit the highest breakdown strength of 1000 MV/m, while the value for PI and PEI films is only 600 MV/m. Even at 200 °C, the breakdown strength of blended films with 50% PEI can still be maintained at 550 MV/m, 35% higher than the pure PI or PEI films (400 MV/m) (Figure 4h). Consequently, the energy density of a PI/PEI blended film with 50% PEI is 8 J/cm^3^, while the energy densities of pure PI and PEI films are below 5 J/cm^3^ (Figure 4i). In addition to PEI, poly(1,4-phenylene ether-sulfone) (PSU) containing the same negatively charged group in its polymer chain was combined with PI. The breakdown strength and energy density of the PI/PSU blended films also show significant improvement compared to that of the PI or PSU films. Therefore, reducing the weak points in dielectric polymers is an effective strategy for enhancing the breakdown strength of dielectric polymers. 

Introducing polymers with high dielectric constants into linear dielectric polymers has been shown to be effective for improving their energy storage performance. As can be seen from Table 1, PEEU exhibits higher permittivity (4.7) and breakdown strength (600 MV/m) [58,59]. Thus, PEEU has attracted an increasing amount of attention. However, the poor flexibility caused by the small molecular weight of PEEU greatly hinders its practical application. To solve this problem, PEEU was introduced into the PI matrix as an “organic filler”. As a result, PEEU/PI composite films with good flexibility and high energy storage performance have been fabricated [60].

**Figure 5:**
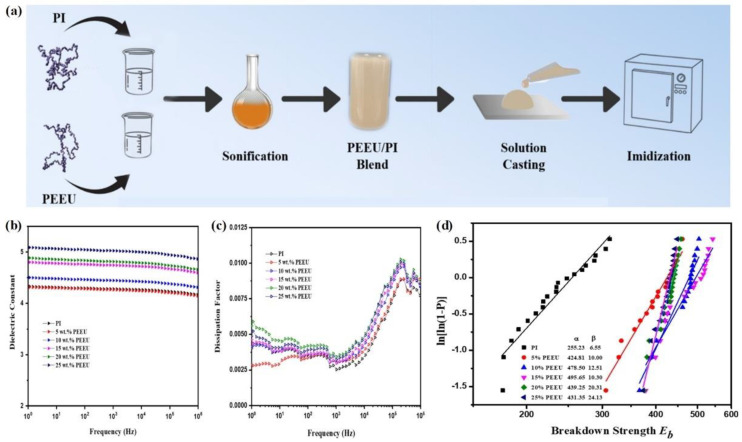
(**a**) Schematics of the synthesis process of PEEU/PI blend films. (**b**,**c**) The dielectric constant (**b**) and dissipation factor (**c**) of PEEU/PI blend films with various ratios as a function of frequency at ambient temperature. (**d**) Weibull breakdown strength of PI and PEEU/PI blend films at ambient temperature (measured at 10^3^ Hz). Reproduced with permission from Ref. [60]. Copyright 2017, Wiley–VCH.

Figure 5a presents the synthesis process for obtaining PEEU/PI blended films. By adjusting the proportion of PEEU and PI, PEEU/PI blended films with ratios of 5/95, 10/90, 15/85, 20/80, and 25/75 were obtained. Figure 5b shows the dielectric constants of PEEU/PI blended films with varying PEEU content. The dielectric constant of the blended films increases with increasing PEEU content in these composite films. In particular, the dielectric constant of the 25/75 PEEU/PI blended film is 5.02, about 17% higher than that of pristine PI (4.28). Meanwhile, all the PEEU/PI blended films exhibit a relatively low dissipation factor during the tested frequency range (Figure 5c). 

Figure 5d presents the Weibull breakdown strength values of PEEU/PI blended films. The shape parameter (*β*) values of all the blended films (ranging from 10.00 to 24.13) are higher than that of the PI film (6.55). Meanwhile, the breakdown strength values of all the PEEU/PI blended films are higher than that of the PI film. In particular, the 15/85 PEEU/PI blended film exhibits a breakdown strength of 495.65 MV/m, almost twice that of pristine PI (255.23 MV/m). Due to the increased breakdown strength and enhanced dielectric constant, the energy density of the 15/85 PEEU/PI film (5.14 J/cm^3^) is more than four times higher than that of PI (1.23 J/cm^3^). 

### 2.4. Ferroelectric/Linear Dielectric Polymer Blends

Compared with linear dielectric polymers, ferroelectric polymers usually exhibit higher energy densities due to their higher permittivity [61,62]. For example, the permittivity of poly(vinylidene fluoride-trifluoroethylene-chlorofluoroethylene) (P(VDF-TrFE-CFE)) is about 50. However, ferroelectric polymers such as PVDF, PVDF copolymers, and PFDF terpolymers often exhibit high dissipation factors (0.02), leading to low charge–discharge efficiency. Recently, blended films prepared with a linear dielectric polymer matrix and high permittivity ferroelectric polymers have attracted global attention, due to their potential for improving the dielectric constant of blended films without sacrificing breakdown strength. 

**Figure 6:**
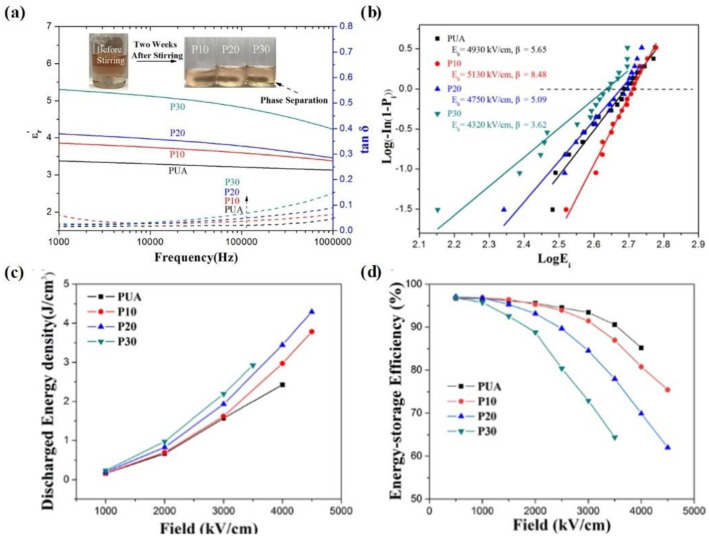
Dielectric constant (**a**), breakdown strength (**b**), discharged energy density (**c**) and energy-storage efficiency of PUA and PUA/P(VDF-TrFE-CFE) blend films (**d**) at ambient temperature. The breakdown strength was tested under a voltage ramp of 0.1 kV s^−1^, and the P-E hysteresis loops were tested at 10 Hz. Reproduced with permission from Ref. [63]. Copyright 2019, IOP.

Zhou et al. significantly improved the energy storage performance of pure polyurea (PUA) film by incorporating (P(VDF-TrFE-CFE)) into a PUA matrix [63]. By adjusting the content of P(VDF-TrFE-CFE) in the P(VDF-TrFE-CFE)/PUA blended solution, various P(VDF-TrFE-CFE)/PUA composite films were obtained. Figure 6a presents the dielectric properties of pure PUA film and various blended films, where P10 represents the composite film with 10 vol % P(VDF-TrFE-CFE). Due to the high dielectric constant of P(VDF-TrFE-CFE), the blended film with 30 vol % P(VDF-TrFE-CFE) exhibits the highest dielectric constant of 5.3 at 10^3^ Hz. Meanwhile, the dielectric loss shows a slightly increasing tendency with increasing P(VDF-TrFE-CFE) content. Zhou et al. further calculated the breakdown strength of pristine PUA and various blended films, as shown in Figure 6b. Compared with the breakdown strength of pristine PUA film (4930 kV/cm), the value of P10 is 5130 kV/cm. However, for blended films with 20 and 30 vol % P(VDF-TrFE-CFE), the breakdown strength significantly decreases to 4750 kV/cm and 4320 kV/cm. 

Figure 6c,d presents the energy storage performance of PUA and various blended films. On the one hand, due to the enhancement of the dielectric constant by P(VDF-TrFE-CFE), the energy density of the blended films increases with increasing P(VDF-TrFE-CFE) content under the same electric field (Figure 6c). On the other hand, the introduction of P(VDF-TrFE-CFE) has a negative influence on the charge–discharge efficiency of the P(VDF-TrFE-CFE)/PUA films, which is mainly due to its high conduction loss. 

Therefore, P(VDF-TrFE-CFE) is beneficial for improving the dielectric constant of blended films. Consequently, the energy density of P(VDF-TrFE-CFE) blended films significantly improves with increasing P(VDF-TrFE-CFE) content under the same electric field (Figure 6c). However, the ferroelectric properties and high field conduction loss of P(VDF-TrFE-CFE) negatively affect the efficiency of these composite films (Figure 6d). As a result, the discharge energy density of the P20 composite film is 4.3 J/cm^3^, while that of pristine PUA is only 2.43 J/cm^3^. Overall, introducing ferroelectric polymers with high permittivity as a functional filler into a linear dielectric polymer matrix has proven to be beneficial for improving the energy-storage performance of linear dielectric polymers.

## 3. Linear Polymer Nanocomposites

### 3.1. The 0D Nanoparticle/Linear Polymer Nanocomposites

#### 3.1.1. Nanoparticles with High Permittivity

In the past decades, many studies dedicated to improving the energy storage properties of linear dielectrics have been reported. Among these, polymer nanocomposite film, which is usually composed of polymer matrix and a nanofiller, has attracted an increasing amount of attention [32,64]. As the second phase of these nanocomposite films, the intrinsic features of the nanofiller, including dielectric constant, shape, size, and concentration, have a great effect on the energy storage performance of these materials. In addition, the polymer/filler interface and the spatial composite structure of the polymer nanocomposites are also highly significant [65,66,67]. Ceramic dielectrics, such as BaTiO_3_ (BTO), TiO_2_, and (Ba_x_Sr_1−x_)TiO_3_, usually have much higher dielectric constants than polymers [68,69,70]. Therefore, incorporating ceramic nanofillers as functional materials into linear polymers is a good strategy for fabricating polymer nanocomposites with improved energy storage performance. 

**Figure 7:**
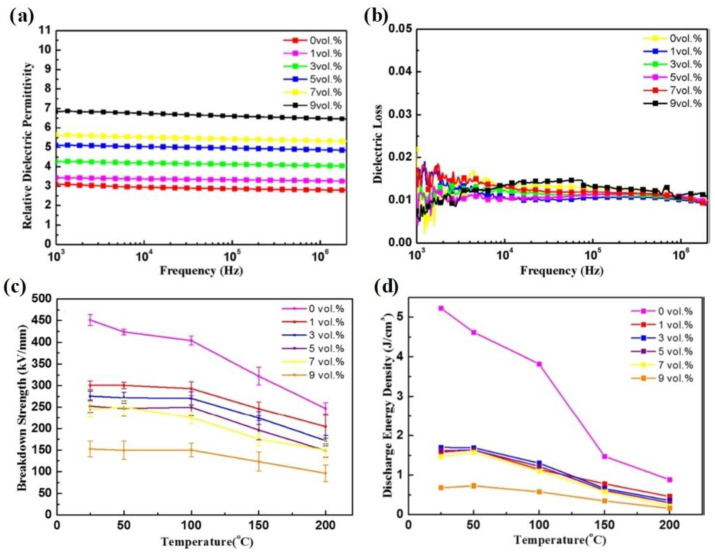
The relative dielectric permittivity (**a**) and dielectric loss (**b**) of BTO/PI nanocomposites with a BTO content of 0, 1, 3, 5, 7, and 9 vol % at ambient temperature. Dependences of breakdown strength (**c**) and discharge energy density (**d**) for pure PI and BTO/PI nanocomposites with BTO content of 1, 3, 5, 7, and 9 vol % on temperature ranging from 25 °C to 200 °C at 100 Hz. Reproduced with permission from Ref. [71]. Copyright 2017, American Institute of Physics.

Sun et al. fabricated BTO/PI nanocomposites with good energy storage performance by incorporating BTO nanoparticles into PI [71]. Figure 7a presents the dielectric constants of BTO/PI nanocomposites as a function of BTO content at room temperature. The results show that the dielectric constant of a nanocomposite with 9 vol % BTO is about 6.8, more than twice that of pure PI (~3.1). This dielectric constant enhancement can be attributed to the following reasons. Firstly, the introduction of BTO nanoparticles with a high dielectric constant increases the permanent dipoles in the nanocomposites. Secondly, the large quantity of interfacial boundaries between the BTO nanoparticles and the PI matrix significantly promotes interfacial polarization in the nanocomposites. As is consistent with the pure PI film, the dielectric loss of all the BTO/PI nanocomposites is almost unchanged in the tested frequency (10^3^ to 10^6^ Hz) (Figure 7b). 

Figure 7c summarizes the breakdown strength of PI and various BTO/PI nanocomposites under different temperatures, varying from 25 °C to 200 °C. The results show that the introduction of BTO nanoparticles has a negative influence on the breakdown strength of the nanocomposites. For instance, the breakdown strength of pure PI film at room temperature is about 450 kV/mm, while the corresponding value for BTO/PI composite film with 9 vol % BTO is only about 150 kV/mm. The significant difference in the permittivity between the nanofiller (BTO nanoparticle) and polymer matrix (PI) may account for the sharp decrease in the breakdown strength of the composite films. Moreover, the structural imperfections induced by the introduction of the nanofiller may also contribute to the decreasing breakdown strength. Due to the sharply decreased breakdown strength, the discharge energy density values of the BTO/PI composite films are lower than that of pure PI (Figure 7d). Therefore, although the introduction of BTO can effectively enhance the permittivity of the composite films, their energy density is not improved. In general, the introduction of nanofillers with high dielectric constants can effectively improve the dielectric constant of composite materials, but this does not improve their energy density. Therefore, to obtain polymer nanocomposites with improved energy density, other factors should be considered, particularly polymer/filler interface compatibility.

#### 3.1.2. Surface-Modified High Permittivity Nanoparticles

As discussed above, the polymer/filler interface could significantly influence the dielectric performance of the composites. Generally, surface modification involves grafting suitable ligands onto the surface of the particles to prevent them from agglomerating and make them compatible with other phases. Therefore, surface modification is an effective method to overcome interfacial energy barriers and reduce nanofiller agglomeration in composite films [72]. To date, various modifiers have been reported to improve the polymer/filler interface compatibility, and this strategy has been shown to effectively improve the energy storage performance of polymer nanocomposites. Dang et al. modified the surface of BTO nanoparticles with a silane coupling agent (KH550) and combined the modified BTO nanoparticles with a PVDF matrix, significantly improving the interface compatibility between the BTO and PVDF [73]. Zhou et al. prepared surface hydroxylated BTO nanoparticles (h-BTO) by dispersing BTO in an aqueous solution of H_2_O_2_, showing that h-BTO/PVDF nanocomposites exhibit improved breakdown strength compared to that of unmodified-BTO/PVDF nanocomposites [74]. Yu et al. reported polyvinylpyrrolidone-modified BTO/PVDF nanocomposite films with an improved dielectric constant (77) and enhanced electric breakdown strength (336 MV/m). The energy density of their polyvinylpyrrolidone-modified BTO/PVDF nanocomposite is 6.8 J/cm^3^ [75]. 

#### 3.1.3. Wide Bandgap Nanoparticles

**Figure 8:**
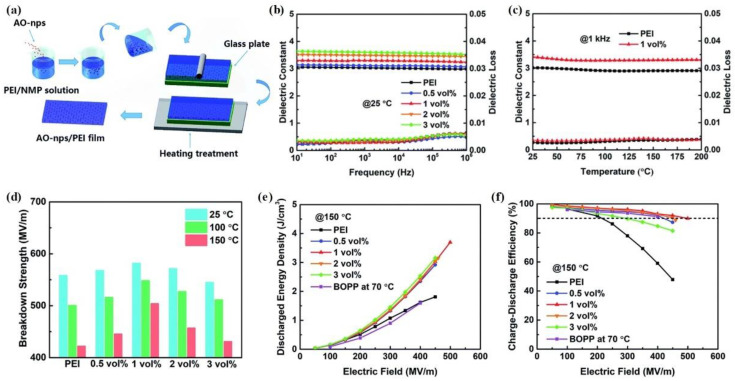
(**a**) Schematic illustration of the fabrication of the Al_2_O_3_/PEI nanocomposites. (**b**) The frequency-dependent dielectric constant of pristine PEI and Al_2_O_3_/PEI nanocomposites at 25 °C. (**c**) Temperature-dependent dielectric constant and loss of pristine PEI and 1 vol % Al_2_O_3_/PEI nanocomposite at 1 kHz. (**d**) Breakdown strength of pristine PEI and Al_2_O_3_/PEI nanocomposites at 25 °C, 100 °C, and 150 °C. (**e**,**f**) Discharge energy density and efficiency of pure PEI and Al_2_O_3_/PEI nanocomposites at 150 °C. Reproduced with permission from Ref. [76]. Copyright 2020, The Royal Society of Chemistry.

Although the surface modification of nanofillers is beneficial for improving the energy density of nanofiller/polymer nanocomposites, complicated and time-wasting preparation processes often limit their industrial application. Recently, several new nanofillers with relatively low permittivity but wide bandgaps have been incorporated with polymer matrices without surface modification and the reported results show that these composites exhibit high energy storage performance. Fan et al. fabricated Al_2_O_3_ nanoparticle/PEI nanocomposite films by simply casting a mixed precursor solution on a glass plate, as shown in Figure 8a. The energy storage properties of blended films as a function of Al_2_O_3_ content at various temperatures were also studied [76]. 

Figure 8b shows the dielectric constant and dielectric loss of Al_2_O_3_/PEI blended films with varying Al_2_O_3_ volume content. Compared with the PEI film, the dielectric constant of the Al_2_O_3_/PEI nanocomposites slightly increases from 3.11 to 3.6 when the volume percentage of Al_2_O_3_ increases from 0.5% to 3%. Meanwhile, the dielectric loss of all the nanocomposites remains below 0.005 over the tested frequency. Moreover, the dielectric properties of the Al_2_O_3_/PEI nanocomposite with 1 vol % Al_2_O_3_ remain stable across the tested temperature range (25 °C to 200 °C), indicating that the Al_2_O_3_/PEI nanocomposites exhibit high thermal stability (Figure 8c). 

Figure 8d presents the breakdown strength of various Al_2_O_3_/PEI nanocomposites at different temperatures. The breakdown strength of these Al_2_O_3_/PEI nanocomposites increases with increasing Al_2_O_3_ content from 0 to 1 vol % at temperatures up to 150 °C. However, when the Al_2_O_3_ content increases to 2 vol % and 3 vol %, the breakdown strength then decreases. This is potentially due to the weak points caused by the aggregation of Al_2_O_3_ nanoparticles. Moreover, due to the high thermal stability of the Al_2_O_3_/PEI composites, the breakdown strengths of the Al_2_O_3_/PEI composites only exhibit a slight decrease with increasing temperature. For the composite film with 1 vol % Al_2_O_3_, the breakdown strength at 150 °C is only about 13% lower than that at 25 °C. However, the breakdown strength values of the PEI films at 150 °C are 24% lower than those at 25 °C. Due to their enhanced dielectric constants, the significantly increased breakdown strength, and the high-temperature stability, the energy storage performance of the Al_2_O_3_/PEI nanocomposites at 150 °C is significantly improved compared with the corresponding PEI film values (Figure 8e,f). Specifically, the recoverable energy density and efficiency of the 1 vol % Al_2_O_3_/PEI nanocomposite at 150 °C are 3.7 J/cm^3^ and 90.1%, respectively, while the corresponding values of the PEI film are only 1.81 J/cm^3^ and 47.9%, respectively. 

### 3.2. The 1D Nanofiber/Linear Polymer Nanocomposites

**Figure 9:**
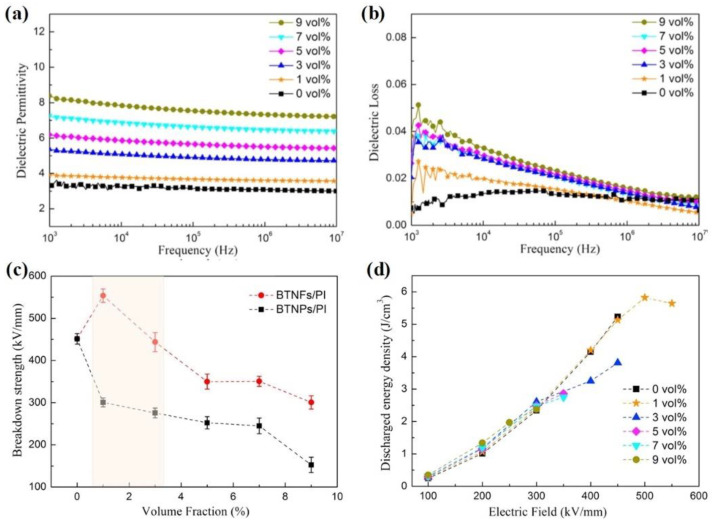
Frequency-dependency of dielectric permittivity (**a**) and dielectric loss (**b**) of BTNFs/PI nanocomposites. (**c**) Breakdown strength of BTNFs/PI and BTNPs/PI nanocomposites as a function of content loading. (**d**) Discharged energy density of BTNFs/PI nanocomposites as a function of the electric field. Reproduced with permission from Ref. [78]. Copyright 2018, Elsevier.

In addition to 0D nanoparticles, 1D nanomaterials (such as nanorods, nanofibers, and nanowires) are also used as composite materials with polymer matrices to enhance the energy storage properties of linear dielectric polymers. Compared with nanoparticles, 1D nanomaterials can significantly improve the breakdown strength of polymer composites, mainly due to their high aspect ratio and small specific surface area [24,77]. Hu et al. fabricated BaTiO_3_ nanofibers (BTNFs) by electrospinning and then compounded the nanofibers with a PI matrix to prepare BTNFs/PI nanocomposites with varying BTNF volume content [78]. As shown in Figure 9a, with the BTNF content increasing from 0 to 9 vol %, the permittivity of the BTNFs/PI nanocomposites significantly increases from 3.1 to 8.3 at 1 kHz. This is mainly due to the high permittivity of the BTNFs. Meanwhile, the dielectric loss of the BTNFs/PI nanocomposites slightly increases with increasing BTNF content, although the dielectric loss of all the nanocomposites is below 0.04 (Figure 9b). 

Figure 9c shows the breakdown strengths of BTNFs/PI nanocomposites and composites made with BT nanoparticles (BTNPs). The breakdown strength of the BTNFs/PI composite films is higher than the value of BTNPs/PI nanocomposites at the same nanofiller volume content. Specifically, the 1 vol % BTNFs/PI nanocomposite exhibits a high breakdown strength of is 553 kV/mm, while the values for PI film and 1 vol % BTNPs/PI nanocomposite are 450 kV/mm and 300 kV/mm, respectively. It is worth noting that when the volume percentage of the nanofiller increases from 3 vol % to 9 vol %, the breakdown strength of both the BTNFs/PI and BTNPs/PI nanocomposites continuously decreases, which can be attributed to the inevitable aggregation of the nanofillers. As a result, the BTNFs/PI nanocomposite with 1 vol % BTNFs exhibits the highest energy density of 5.83 J/cm^3^ under an electric field of 500 kV/mm, slightly better than that of the pure PI film (Figure 9d). Therefore, although 1D nanofillers can enhance the breakdown strength of polymer-based composites, the dispersion of these 1D nanofillers at high concentrations must be enhanced in order to significantly improve the energy density of the composites.

### 3.3. The 2D Nanoplate/Linear Polymer Nanocomposites

As previously discussed, the structural continuity and thermal conductivity of nanofillers are of great importance to the energy storage performance of polymer-based nanocomposites. Therefore, emerging 2D nanomaterials may provide new possibilities for the fabrication of high-performance dielectric capacitors [79,80,81,82]. Compared with 1D nanomaterials, 2D fillers with high aspect ratios can establish effective conductive barriers in nanocomposites to hinder the growth of electrical trees during the breakdown process and increasing breakdown strength [23]. In addition, the high thermal conductivity of 2D materials can effectively dissipate Joule heat, which is beneficial for enhancing the high-temperature performance of dielectrics.

**Figure 10:**
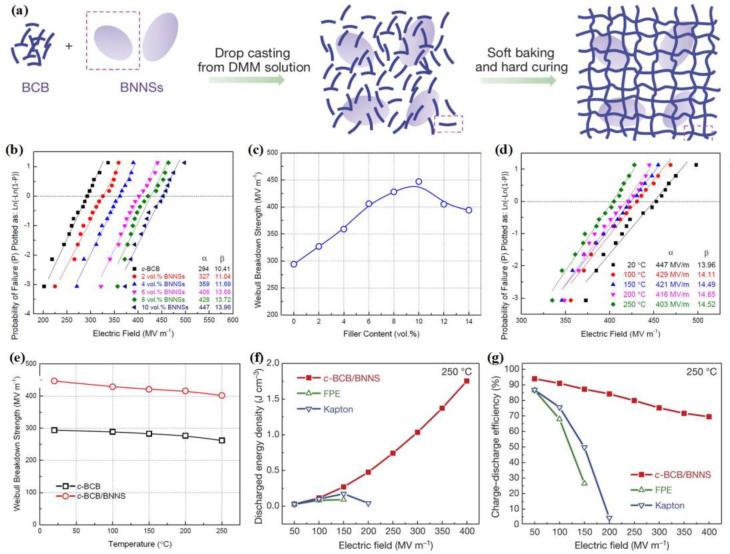
(**a**) Schematic of the preparation of *c*-BCB/BNNS films. (**b**,**c**) Weibull breakdown strength of *c*-BCB/BNNS as a function of the BNNSs content. (**d**) Weibull plots of *c*-BCB/BNNS with 10 vol % of BNNSs at different temperatures. (**e**) Weibull breakdown strength of *c*-BCB and *c*-BCB/BNNS as a function of temperature. The dielectric breakdown strength was measured using the electrostatic pull-down method under a direct-current voltage ramp of 500 V s^−1^. (**f**,**g**) Discharged energy density and efficiency of *c*-BCB/BNNS with 10 vol % of BNNSs at 250 °C (Measured at a frequency of 10 Hz). Reproduced with permission from Ref. [40]. Copyright 2015, Springer Nature.

Li et al. prepared a 2D polymer nanocomposite by dispersing boron nitride nanosheets (BNNSs) in the high *T*_g_ divinyltetramethyldisiloxane-bis(benzocyclobutene) (BCB) matrix, followed by thermal crosslinking. As a result, crosslinked-BCB (*c*-BCB)/BNNSs dielectric nanocomposites with stable cross-linking networks and BNNSs insulation networks were obtained (Figure 10a) [40]. Figure 10b,c summarizes the breakdown strength of the (*c*-BCB)/BNNSs nanocomposites. A high breakdown strength of 447 MV/m is achieved when incorporating 10 vol % BNNSs into the *c*-BCB matrix. The improvement in the breakdown strength of the (*c*-BCB)/BNNSs nanocomposites is attributed to the improvement of Young’s modulus and the effective suppression of high-field electrical conduction by the introduction of BNNSs. In addition, the ultrahigh thermal conductivity of BNNSs (~300 to 2000 W/(m/K)) is conducive to improving the breakdown strength at high temperatures. Specifically, the breakdown strength of 10 vol % (*c*-BCB)/BNNSs at 250 °C is 403 MV/m, only 9.8% lower than that at 20 °C (Figure 10d). In contrast, the breakdown strength of the pristine *c*-BCB film at 250 °C sharply decreases to ~260 MV/m (Figure 10e). 

Figure 10f,g shows the energy density and efficiency of composite films with 10 vol % BNNSs and two other high-temperature-resistant dielectrics at 250 °C. Remarkably, the energy density of the 10 vol % (*c*-BCB)/BNNSs composite film is 1.8 J/cm^3^ at a relatively high electric field of 400 MV/m, while fluorene polyester (FPE) and Kapton can only operate under low electric fields of 150 MV/m and 200 MV/m with ultralow discharge energy density. In addition, the efficiencies of FPE and Kapton sharply decrease from ~80% to less than 30% when the electric field increases to 150 MV/m, while the efficiency of (*c*-BCB)/BNNSs is still higher than 70% at 400 MV/m. 

According to Li et al., the energy-storage performance of polymer nanocomposites is effectively improved by incorporating BNNSs into polymer matrices, especially for high-temperature operation. However, the typical BNNSs preparation method, liquid-phase exfoliation, is both time-consuming and low-yield, which greatly hinders the scale-up of BNNSs production [83]. In addition to BNNSs, *γ*-Al_2_O_3_ is another typical wide bandgap material, and its dielectric constant (9–10) is higher than that of BNNSs (3–4). Moreover, *γ*-Al_2_O_3_ usually exhibits a high breakdown strength of 600–800 MV/m. Therefore, *γ*-Al_2_O_3_ is an ideal nanofiller for the fabrication of high-performance polymer nanocomposites. 

**Figure 11:**
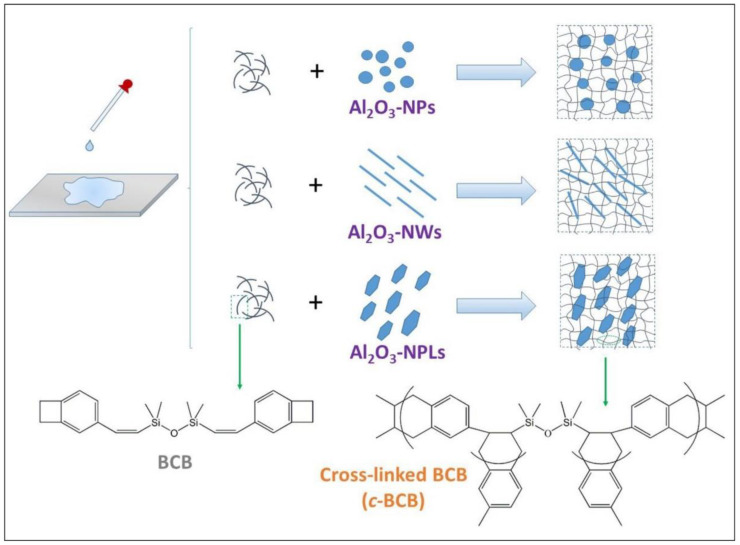
Schematic of the preparation of *c*-BCB/Al_2_O_3_-NPs, -NWs and -NPLs nanocomposites. Reproduced with permission from Ref. [84]. Copyright 2019, Wiley–VCH.

**Figure 12:**
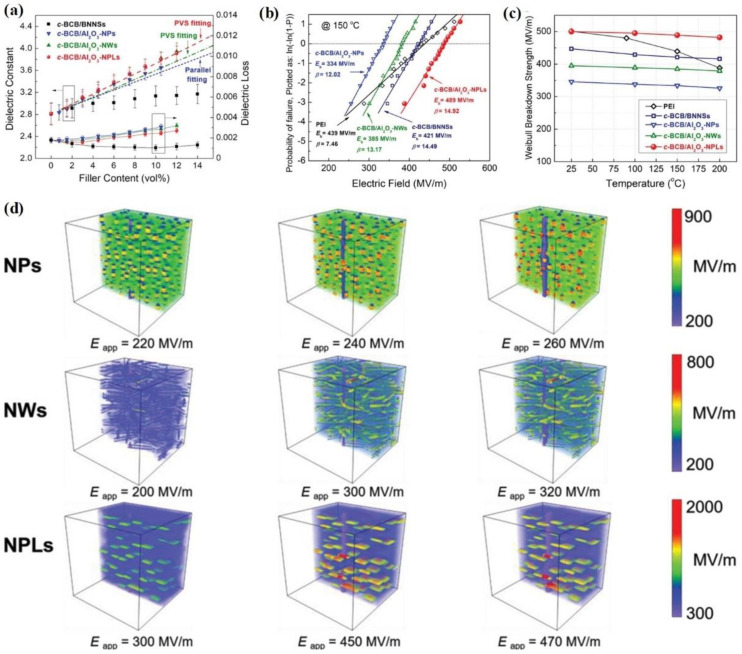
(**a**) Dielectric constant and loss of the composites as a function of filler content measured at room temperature and 1 kHz. (**b**) Weibull statistic of dielectric breakdown strength of PEI and the nanocomposites at 150 °C. (**c**) Temperature-dependent Weibull breakdown strength of PEI and the nanocomposites at 150 °C. (**d**) The corresponding electric field distribution was computed by phase-field simulations of the *c*-BCB nanocomposites with 7.5 vol % Al_2_O_3_ NPs, NWs, and NPLs at 150 °C and varied applied electric fields. Reproduced with permission from Ref. [84]. Copyright 2019, Wiley–VCH.

Based on this, Li et al. prepared various *c*-BCB-based composites by introducing Al_2_O_3_ nanoparticles, Al_2_O_3_ nanowires, and Al_2_O_3_ nanoplates as fillers, denoted *c*-BCB/Al_2_O_3_-NPs, *c*-BCB/Al_2_O_3_-NWs, and *c*-BCB/Al_2_O_3_-NPLs, respectively (Figure 11) [84]. Figure 12a presents the dielectric constants of the Al_2_O_3_-based polymer composites and the *c*-BCB/BNNSs composite reported in [40]. All the reported Al_2_O_3_-based polymer composites exhibit higher dielectric constants than the *c*-BCB/BNNSs composite at the same filler content, due to the relatively high permittivity of Al_2_O_3_. Moreover, compared with *c*-BCB/Al_2_O_3_-NPs, the dielectric constants of *c*-BCB/Al_2_O_3_-NWs and *c*-BCB/Al_2_O_3_-NPLs with the same nanofiller content are much higher, which can be attributed to the larger dipole moments caused by the higher aspect ratios of Al_2_O_3_-NWs andAl_2_O_3_-NPLs.

The breakdown strength of various dielectric nanocomposites at 150 °C is shown in Figure 12b. On one hand, nanocomposites containing 2D nanofillers (*c*-BCB/Al_2_O_3_-NPLs and *c*-BCB/BNNSs) exhibit higher breakdown strengths than nanocomposites containing 0D Al_2_O_3_ nanoparticles (*c*-BCB/Al_2_O_3_ NPs) and 1D Al_2_O_3_ nanowires (*c*-BCB/Al_2_O_3_ NWs). On the other hand, compared with the nanocomposite containing 10 vol % BNNSs, the 7.5 vol % Al_2_O_3_-NPLs nanocomposite has a higher breakdown strength (489 MV/m vs. 421 MV/m). Moreover, the breakdown strength temperature stability of all the Al_2_O_3_-based polymer composites is higher than that of pure PEI film (Figure 12c). Specifically, the breakdown strength of *c*-BCB/Al_2_O_3_ NPLs is almost constant over a wide temperature range of 25 °C to 200 °C, while a significant decrease of 20% can be observed for PEI film (from 501 to 400 MV/m).

To further understand the breakdown strength enhancement of Al_2_O_3_-based polymer composites, phase-field simulations of various composites were characterized, as shown in Figure 12d. For the *c*-BCB/Al_2_O_3_ NPs and the *c*-BCB/Al_2_O_3_ NWs composites, the electric fields are highly concentrated around the NPs and at the vertices of the NWs, respectively, which leads to the easy formation of breakdown paths. In contrast, the NPLs effectively disperse the applied electric field through the polymer matrix to mitigate the inhomogeneous distribution of local electric fields, resulting in higher breakdown strength. 

**Figure 13:**
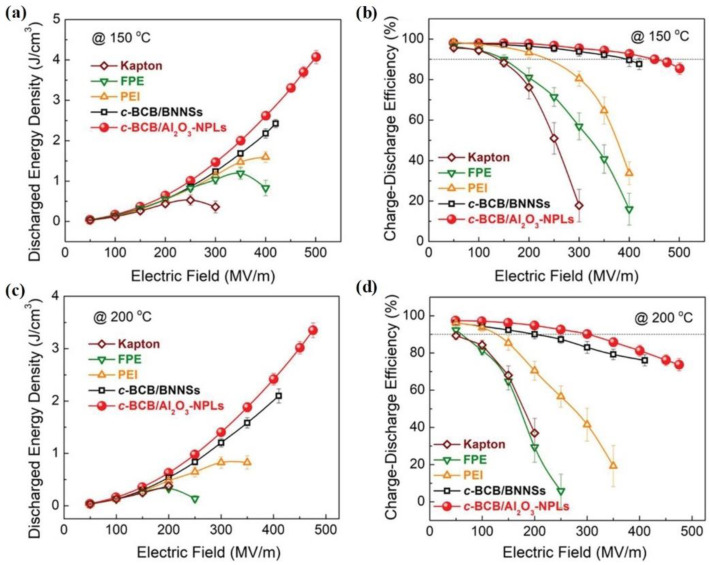
Discharged energy density and charge–discharge efficiency of high-temperature dielectric polymers and the *c*-BCB nanocomposites measured at 150 °C (**a**,**b**) and 200 °C (**c**,**d**). Reproduced with permission from Ref. [84]. Copyright 2019, Wiley–VCH.

Due to its high dielectric constant and the excellent temperature stability of its breakdown strength, *c*-BCB/Al_2_O_3_ NPLs exhibit better high-temperature energy storage properties than current high-temperature dielectrics. As shown in Figure 13a, the energy density of *c*-BCB/Al_2_O_3_-7.5 vol % NPLs at 150 °C is 4.3 J/cm^3^, while typical values for other high-temperature dielectrics are below 2.5 J/cm^3^. Moreover, the efficiency of *c*-BCB/Al_2_O_3_ NPLs remains > 90% under a high electric field of 450 MV/m (Figure 13b). Even at 200 °C, the energy storage performance of *c*-BCB/Al_2_O_3_ NPLs is still much better than that of other dielectrics. For instance, the energy density of *c*-BCB/Al_2_O_3_ NPLs is about 3.02 J/cm^3^ and the efficiency is > 75% at 450 MV/m (Figure 13c,d). 

## 4. Conclusions and Future Perspective

In summary, compared with ferroelectrics, relaxor ferroelectrics, and anti-ferroelectrics, linear dielectric polymers exhibit obvious advantages in charge–discharge efficiency but usually suffer from low energy density. Therefore, the energy density of linear polymer dielectrics must be significantly improved to realize their practical application in electrical power systems. To date, various methods have been developed to effectively improve the energy storage performance of linear dielectrics. This improvement is mainly achieved in one of two ways: increasing the breakdown strength and enhancing the dielectric constant. In terms of increasing the breakdown strength of linear polymers, preparing polymer blends has proven to be effective for reducing the weak points in polymers. In addition, incorporating high-permittivity nanofillers with linear polymer matrices and controlling polymer/filler interfaces can improve the permittivity of polymer composites while ensuring that the breakdown strength does not significantly decrease. In this way, energy density is enhanced. 

Various linear polymer nanocomposites with improved energy storage performance have been obtained. By compounding wide bandgap Al_2_O_3_ nanoparticles with a PEEU matrix, a discharge energy density of up to 27 J/cm^3^ can be obtained at room temperature with the resulting Al_2_O_3_/PEEU composite, which has a charge-discharge efficiency of > 90% [43]. The high-temperature energy storage performance of linear dielectrics has also been significantly improved. The incorporation of 2D Al_2_O_3_ nanoplates with a BCB matrix results in a nanocomposite with an energy density of 3 J/cm^3^ at 200 °C. More importantly, the efficiency of this nanocomposite is > 75% at this temperature [84]. However, despite the substantial amount of progress made in improving the properties of linear polymer dielectrics, these dielectrics still fall short of the requirements for their desired applications, and the future development of linear dielectrics should include the following aspects:

1. More attention should be paid to the chemical or physical modification of the molecular structures and nanostructures of linear dielectric polymers to improve their saturation polarization levels and breakdown strength. This is the most fundamental and effective way to improve the energy density of linear dielectric polymers without sacrificing their efficiency. 

2. The polarization level of linear dielectric polymers can be effectively improved by the introduction of high-permittivity nanofillers but their breakdown strength will be correspondingly reduced, due to the inevitable structural defects caused by the nanofiller phase. Therefore, innovative approaches should be developed to improve the dispersion uniformity of nanofillers and to optimize polymer/filler interface compatibility.

3. The conduction loss of polymer nanocomposites usually sharply increases with increasing electric field and temperature, which results in a significant decrease in charge-discharge efficiency. To suppress this temperature- and field-dependent conduction loss, surface functionalization and molecular engineering should be considered to further optimize polymer dielectric materials. 

4. Finally, the development of feasible, low-cost, and scaled-up manufacturing processes is critical for the industrial application of dielectric materials. This endeavor mainly includes two aspects: the simplification of the existing dielectric material preparation approaches and the development of large-scale production apparatus. 

## Figures and Tables

**Table 1:** Summary of basic properties of several linear dielectric polymers.

Polymer	Max Operating Temperature (°C)	Permittivity (at 1 kHz)	Dissipation Factor (at 1 kHz) (%)	Breakdown Strength (MV/m) (Film Thickness in Parentheses)
BOPP	105	2.2	<0.02	449–555 (3–5 μm)
PI (Kapton)	360–410	2.7–3.5	0.13–0.26	154–303 (7.6–127 μm)
PI (UPILEX)	285–500	3.2–3.5	0.13–0.7	147–320 (12.5–125 μm)
PEI (ULTEM)	217–247	3.15	0.12	200 (25 μm)
LDPE	95–113	3	<0.05	200 (30 μm)
PEEU	250	4.7	<1	600 (2–3 μm)
PMMA	150	3.3	<5	550 (13–24 μm)
PET	125	3.3	<0.5	570 (3 μm)
PC	125	2.9	<0.2	550 (0–13 μm)
